# Central nervous system macrophages in progressive multiple sclerosis: relationship to neurodegeneration and therapeutics

**DOI:** 10.1186/s12974-022-02408-y

**Published:** 2022-02-10

**Authors:** Emily Kamma, Wendy Lasisi, Cole Libner, Huah Shin Ng, Jason R. Plemel

**Affiliations:** 1 Department of Pathology and Laboratory Medicine, Faculty of Medicine, University of British Columbia, Vancouver, BC Canada; 2 Recovery and Performance Laboratory, Faculty of Medicine, Memorial University of Newfoundland, Saint John’s, NL Canada; 3 Department of Health Sciences and the Office of the Saskatchewan Multiple Sclerosis Clinical Research Chair, College of Medicine, University of Saskatchewan, Saskatoon, SK Canada; 4 Division of Neurology and the Djavad Mowafaghian Centre for Brain Health, Department of Medicine, University of British Columbia, Vancouver, BC Canada; 5 Division of Neurology, Department of Medicine, University of Alberta, Edmonton, AB Canada; 6 Neuroscience and Mental Health Institute, University of Alberta, Edmonton, AB Canada; 7 Department of Medical Microbiology and Immunology, University of Alberta, Edmonton, AB Canada; 8 University of Alberta, 5-64 Heritage Medical Research Centre, Edmonton, AB T6G2S2 Canada

**Keywords:** Macrophages, Microglia, Multiple sclerosis, Neurodegeneration, Primary progressive, Secondary progressive, Therapeutics

## Abstract

There are over 15 disease-modifying drugs that have been approved over the last 20 years for the treatment of relapsing–remitting multiple sclerosis (MS), but there are limited treatment options available for progressive MS. The development of new drugs for the treatment of progressive MS remains challenging as the pathophysiology of progressive MS is poorly understood.

The progressive phase of MS is dominated by neurodegeneration and a heightened innate immune response with trapped immune cells behind a closed blood–brain barrier in the central nervous system. Here we review microglia and border-associated macrophages, which include perivascular, meningeal, and choroid plexus macrophages, during the progressive phase of MS. These cells are vital and are largely the basis to define lesion types in MS. We will review the evidence that reactive microglia and macrophages upregulate pro-inflammatory genes and downregulate homeostatic genes, that may promote neurodegeneration in progressive MS. We will also review the factors that regulate microglia and macrophage function during progressive MS, as well as potential toxic functions of these cells. Disease-modifying drugs that solely target microglia and macrophage in progressive MS are lacking. The recent treatment successes for progressive MS include include B-cell depletion therapies and sphingosine-1-phosphate receptor modulators. We will describe several therapies being evaluated as a potential treatment option for progressive MS, such as immunomodulatory therapies that can target myeloid cells or as a potential neuroprotective agent.

## Background

Multiple sclerosis (MS) is a chronic immune-mediated disease characterized by inflammation, demyelination, gliosis, and neurodegeneration in the central nervous system (CNS) [1]. It is estimated that over 2.8 million people are living with MS worldwide [2] and the prevalence of MS is projected to increase over time [3]. There are over 15 disease-modifying drugs (DMDs) approved over the last 20 years for the treatment of relapsing–remitting MS (RRMS), which is how about 85% of the MS population initially presents [4, 5]. However, there is only limited treatment choices available for the remaining 10–15% of people with primary progressive MS (PPMS) [4, 5]. Of those who present with RRMS at diagnosis, approximately 50–80% will continue to develop secondary progressive MS (SPMS) within one to two decades [4–6]. Unlike RRMS, the treatment options for SPMS are limited and are mainly restricted to those with active phase of SPMS characterized by new magnetic resonance imaging (MRI) activity and relapses; clinical trials of DMDs failed to show beneficial effects in non-active progressive disease [5].

The development and discovery of new drugs for the treatment of progressive MS (PPMS and SPMS) remains challenging due to several reasons. The pathophysiology of progressive MS remains poorly understood [7]. While the experimental autoimmune encephalomyelitis (EAE) model is widely used to study the immunopathogenesis of MS, this model has limited predictive potential for identifying therapies for progressive MS [8–10]. Microglia and macrophages are found to be present in all MS lesions regardless of the phenotypes [8], and many mechanisms may define their contribution to neurodegeneration [11]. The neurodegenerative roles of microglia and macrophages during the pathogenesis of MS is complicated. For example, these cells are also required for the regeneration of lost myelin, a process known as remyelination [12–14]. Several DMDs have been shown to exert some modest effects either indirectly or directly on macrophages or microglia, but no drugs for MS yet targets solely these innate immune cells [11, 15, 16].

In this review, we described the clinical aspects of progressive MS and the roles of microglia and macrophages in progressive MS and neurodegeneration. We also described the types of DMDs approved for progressive MS and the potential treatments in which there is involvement of microglia and macrophages.

## Clinical aspects of progressive MS

### Clinical course of MS

MS results in motor, sensory and cognitive deficits and is one of the leading causes of neurological disability in young adults [1, 17]. The MS disease course is heterogenous, with each person with MS experiencing different symptoms of various severities. MS is diagnosed with the criteria of clinical attacks, dissemination of lesions in space and time and the presence of cerebrospinal fluid (CSF)-specific oligoclonal bands. The clinical course of MS falls into four main categories: clinically isolated syndrome (CIS), RRMS, SPMS and PPMS [18, 19]. The RRMS, SPMS and PPMS phenotypes can be further classified based on two disease modifiers that assess disease activity and progression [20].

MS often presents with a monophasic clinical episode known as CIS, as many as 60–80% of those with MRI detected lesions go on to be clinically diagnosed with MS [21]. Roughly 85% of people with MS (PwMS) present with RRMS, characterized by disease activity of clinical relapses and MRI activity of gadolinium (Gd)-enhancing lesions or new or enlarging T2 lesions, followed by total recovery or partial recovery with periods of stability in between relapses [20, 22, 23]. People with RRMS who have disease activity consisting of relapses or MRI activity are considered ‘RRMS-active’, while those without disease activity are considered ‘RRMS-not active’ [20]. In 50–80% of people diagnosed with RRMS, there is a transition into SPMS within 10–20 years from initial diagnosis [4–6]. The average age of onset for RRMS is 30 while it is between 40 and 50 for progressive MS [24, 25]. SPMS is typically diagnosed retrospectively as there is no clear criteria to determine when RRMS transitions to SPMS [20, 26]. People with SPMS can be considered SPMS-active or SPMS-not active based on the presence of clinical attacks and MRI activity. In addition to disease activity, people with SPMS can be considered ‘SPMS-progressing’ or ‘SPMS-not progressing’ based on clinical evidence of disease progression or confirmed accumulation of disability independent of relapses [20]. While women are typically affected with RRMS and SPMS more than men, with an approximate ratio of 3:1, PPMS affects both sexes equally [27, 28]. About 15% of PwMS are diagnosed with PPMS, presenting with progressive disability from the onset with no clear relapses and minimal to no recovery [22, 23, 29]. Similar to SPMS, people with PPMS can be considered ‘PPMS-active’, ‘PPMS-not active’, ‘PPMS-progressing’ or ‘PPMS-not progressing’ based on assessment of disease activity and progression [20]. Taken all together, people with progressive MS can be classified as ‘active with progression’, ‘active without progression’, ‘not active but with progression’, and finally, ‘not active and without progression’ [20]. Due to recent irregularities on how these terms are used by regulatory authorities, it is recommended that while ‘progressing’ is used to describe accumulated disability independent of relapses, ‘worsening’ should be used to describe any resulting increase in disability or impairment from relapses or increase in disability in the progressive phase [19].

The classification of the MS disease course is dynamic and is constantly revised, particularly to address the confusion that arises with the approval of new DMDs and updated regulations [19]. There are certain issues yet to be addressed with the current classification of MS disease courses. For example, RRMS and SPMS are widely used in clinical practice and research, yet there is no clear distinction between them as the conversion to SPMS is a process that occurs over several years and is only realized retrospectively [23, 26]. Some studies have shown that there is roughly a 3-year period of diagnostic uncertainty during the conversional phase from RRMS to SPMS [30, 31]. In addition, some people with RRMS exhibit progressive features of the disease and some people with progressive forms exhibit relapses and new MRI activity. While relapses predominate in RRMS, clinically silent lesions occur during progressive MS, suggesting that lesion formation is not restricted to RRMS [32, 33]. Therefore, it is unclear if RRMS, SPMS and PPMS are distinct types of MS, or if MS is a disease with a spectrum [23, 24, 34].

The current subtypes of MS are useful for clinical practice and research, but emerging models to classify MS subtypes are being developed to predict disability and relapse rate. These models, which include a topographical model and an MRI abnormality model, could be particularly useful for grouping PwMS during clinical trials. The topographical model uses a real-time simulation software environment to define the dynamic changes of five different factors during the course of MS: lesion localization, relapse frequency, relapse severity, relapse recovery, and baseline brain volume and progression rate [34, 35]. In this model, the CNS contains finite functional reserve, which is lowered during the course of MS. As functional reserve declines, lesions present in clinically silent regions become uncovered. More work is required to identify the neurological substrate of functional reserve and how it is lost in progressive MS.

Eshaghi et al. proposed three new subtypes of MS based on MRI abnormalities including changes in grey matter, normal appearing white matter (NAWM), and lesion load. These subtypes are termed cortex-led, NAWM-led and lesion-led [36]. Compared to cortex or NAWM-led, those categorized as lesion-led were shown to have higher disease activity, greater risk of confirmed disability progression, and improved response to treatment in people with progressive MS. These considerations may be beneficial during clinical trials to stratify participants or to predict disease activity, disability progression, and treatment response. They are also foundations for developing validated models to be used as prognostic and therapeutic guides for individual patients.

### Inflammation and demyelination in MS

MS is a disease largely dominated by inflammation and demyelination that causes tissue damage in the CNS. Although these processes are present in the early and progressive phases of MS, they can vary in severity [25]. The earlier phase of MS is dominated by a peripheral immune response characterized by the infiltration of lymphocytes into the parenchyma—the CNS tissue—through a disrupted blood–brain barrier (BBB) leading to the formation of new active lesions [24, 25]. In contrast, the progressive phase of MS is characterized by the slow expansion of pre-existing lesions, and by a heightened innate CNS immune response with trapped immune cells behind a closed BBB or CSF brain barrier [7, 37, 38]. The integrity of the BBB can be assessed using MRI to detect uptake of Gd-based contrast agents that are administered into the blood prior to imaging [39]. These Gd-enhancing lesions decline until they can no longer be detected as people enter the progressive stages of MS [40]. At this point inflammation is compartmentalized in the CNS and it is thought that expanding lesions add new cortical demyelination and damage to NAWM and normal appearing grey matter (NAGM) [24, 38], with new demyelination potentially occurring around CNS barriers such as the meninges. In addition to inflammation being more focal in the earlier phase of MS and diffuse in the progressive phases, inflammatory activity is more prominent in SPMS than PPMS as there is higher lesion cellularity and more perivascular cuffs in SPMS [23, 41].

In progressive MS, lymphocytic infiltrates consisting of T and B lymphocytes, plasma cells, and macrophages form lymphoid follicles in the CNS [32, 37, 39, 42]. Lymphoid follicles, which are associated with more severe microglia activation and cortical demyelination, are found in large aggregates in the meninges and the perivascular Virchow–Robin spaces [24, 38]. They are typically found in 40–70% of people with SPMS, but not in people with PPMS; however, increased meningeal inflammation associated with more extensive cortical demyelination and neurite loss is present in PPMS, but without lymphoid follicles [43]. Meningeal-associated cortical lesions, or subpial lesions, are prominent in progressive MS [32, 38]. The formation of subpial lesions, often within layers I–IV [41, 44], are thought to be linked to meningeal inflammation based on their close proximity [42, 44]. This pattern is also reproduced by in vivo MRI data and correlates with present and future disability [45]. PwMS with more cortical demyelination and lymphoid follicles have a more rapid MS onset, progression, and death [44, 46, 47].

While demyelination increases with disease duration, remyelination in PwMS is highly variable and declines with increase in age and disease duration [48]. For PwMS, a higher rate of remyelination is associated with lower disability progression [23, 49–51]. Remyelinated lesions in MS typically have thin myelin sheaths and short internodes, making them paler than normal, which is why they are referred to as shadow plaques [23, 52, 53]. In RRMS, shadow plaques are elevated in people younger than 55 or within the first 10 years of diagnosis [48, 51] compared to progressive MS where they are relatively sparse [23, 52, 54, 55].

## CNS macrophages and progressive MS

Microglia and macrophages serve central roles in all stages of MS, and they are required for the initiation of experimental autoimmunity in animal models [56, 57]. These cells are so fundamental to MS disease, that they are largely the basis to define lesion types in MS pathological specimens [58]. While microglia and macrophages generally have a more pathogenic phenotype in the MS CNS, the loss of critical homeostatic functions also potentially contribute to increased damage and reduced repair in progressive MS. In the healthy CNS, homeostatic functions in microglia include secreting growth factors that promote neuronal survival, surveilling the CNS to detect pathogens or tissue injury, and remodeling synaptic connections during development or following injury [59–65]. After demyelination, microglia and macrophages also regulate remyelination [12, 13, 66, 67]. The role of remyelination has been reviewed elsewhere [51, 68, 69]. In this section we described these cells and their involvement thus far in progressive MS.

### Types of CNS macrophages

The normal adult brain contains four types of resident mononuclear phagocytes collectively called CNS macrophages. CNS macrophages include microglia that are located throughout the brain parenchyma and three types of CNS border-associated macrophages (BAMs) that are located at the interface between the CNS and BBB [70]. BAMs consist of perivascular macrophages in the perivascular space between the endothelial and parenchymal basement membranes, meningeal macrophages that line the meninges and its vasculature, and choroid plexus macrophages within the choroid plexus [70–72]. It should be noted that some research groups refer to BAMs as CNS-associated macrophages (CAMs) [73]. Despite their anatomical differences, there are similarities in the development of microglia and BAMs [74]. Fate-mapping studies in mice have found microglia and BAMs largely originate from embryonic progenitors in the prenatal yolk sac that migrate to the brain for maturation [70, 75–77]. Postnatal microglia and BAMs in mice are long-lived and self-renewing cells. Microglia, meningeal macrophages, and perivascular macrophages do not rely on circulating bone marrow-derived hematopoietic progenitors to replenish their population [70, 75, 78]. Only choroid plexus macrophages are partially replenished by monocytes [70, 77].

### Classification and activation of CNS macrophages

Among the CNS macrophages, the roles of microglia are the most well-characterized. In a surveilling state in the healthy CNS, microglia have small cell bodies with a complex highly branched ramified morphology. In a neuroinflammatory environment such as MS, microglia become reactive, a process that includes transcriptional, biochemical, and metabolic remodeling to take on new inflammatory functions [79–81]. Reactive microglia swell and develop rounder cell bodies, taking on a simpler branching pattern with shorter and thicker cell processes [82, 83]. Microglia reactivity includes downregulation of many homeostatic genes, suggesting that there is an impairment and loss of critical homeostatic functions during MS that may worsen neurodegeneration during the pathophysiology of progressive MS [79, 84–86]. There are several key genes that regulate microglial function. For example, *Trem2* is a regulator of phagocytosis and chemotaxis, which are important defensive responses to inflammation and injury in MS [86, 87]. TREM2 is highly expressed by myelin-rich phagocytes in lesions of people with MS, and TREM2 agonists promote myelin debris clearance and enhance remyelination in an animal model of demyelination, suggesting TREM2 activation may be a promising therapeutic avenue in progressive MS [88]. Related to *Trem2* is *Apoe*, a gene critical for lipid metabolism following microglial uptake of myelin lipid debris. *Apoe* expression is upregulated in microglia isolated from mice with EAE and is correlated with disease progression [79]. Activation of the TREM2–APOE pathway prevents the ability of microglia to regulate CNS homeostasis in EAE [79]. Expression of *Cx3cr1*, a homeostatic gene that encodes fractalkine receptor on microglia involved in synaptic pruning and modeling, is also lost in EAE [79]. Blocking the CX3CR1–fractalkine interaction induces microglial production of tumor necrosis factor (TNF)-α, a pro-inflammatory cytokine, and 8-isoprotane, a marker of oxidative stress [89]. Overall, while it is likely true that reactive microglia and macrophages with more pro-inflammatory phenotypes have large contributions to neurodegenerative processes during MS, the failure of microglia and macrophages to maintain reparative and homeostatic functions is also likely important to perpetuate neurodegeneration during progressive disease. Reactive microglia and macrophages express very similar molecular markers, and in many experimental conditions they are indistinguishable [90]. For this reason, the blanket term microglia and macrophage is used, which could also include BAMs.

Because microglia are highly dynamic, they transition through many intermediate morphological forms as they carry out reparative or pathogenic functions [82]. The response of microglia and macrophages to CNS disruption is diverse, but present during virtually all neurological conditions [91]. Upon sensing CNS disruptions such as tissue damage or pathogenic infiltrations, microglia become rapidly reactive. When the disruption is localized, they extend processes to sites of damage [61, 92]. The extension of microglia towards a focal injury can occur in a P2Y12 receptor-dependent manner, and it is in part regulated by extracellular adenosine triphosphate that is enriched after tissue injury [93, 94]. In this neuroprotective response, cell processes rapidly branch out to create a shield surrounding the injury site where they phagocytose pathogens and apoptotic or damaged cell debris [95, 96].

Microglia express over a thousand receptors, and likely respond to hundreds if not thousands of molecules that may drive their reactivity [97]. For example, microglia can also become reactive after toll-like receptor (TLR) or nucleotide-binding oligomerization domain-like receptor-mediated recognition of damage-associated molecular patterns that are released following cellular injury and death in the CNS [98, 99]. This reactivity helps induce the inflammatory cascades that may perpetuate progressive neurodegenerative mechanisms in MS [100]. Under certain circumstances, microglia and macrophages may also contribute to progressive neurodegeneration in MS by producing glutamate, proteases, and reactive oxygen and reactive nitrogen species (ROS/RNS) that may lead to demyelination, neuronal loss, and axonal and mitochondrial damage in lesions (discussed below) [101–105]. Reactive microglia and macrophages also produce inflammatory cytokines such as TNF-α, interleukin (IL)-6, IL-1β, and IL-23 that leads to further immune activation [104, 106–109]. People with progressive MS have elevated levels of these cytokines within blood serum, CSF, and CNS lesions compared to people with RRMS or healthy controls [110–112].

Microglia and macrophages are also necessary for remyelination and may have several other neuroprotective roles [12, 14, 68]. Microglia and macrophages help recruit and maintain oligodendrocyte progenitor cells (OPCs) and their ability to myelinate neurons [66, 67, 113]. They secrete neurotrophic factors including insulin-like growth factor-1 that are required for OPC survival and differentiation into oligodendrocytes [114–117]. In mice with EAE, microglia can also phagocytose and kill CNS-infiltrating Th17 cells [118].

The dichotomous functions of microglia and macrophages have complicated the literature for decades. Protective and toxic functions were initially ascribed to different polarization states of microglia and macrophages, the pro-inflammatory M1 and immunoregulatory M2 states [119]. Indeed, using transgenic mice with *Nos2* based pro-inflammatory states and *Arg1* immunoregulatory states, Locatelli et al. examined these states during EAE. They found that cells could preferentially express either *Nos2* or *Arg1*, with the most *Nos2*-enriched cells found prior to and at onset of disease inflammation [120]. Given that studies found microglia and macrophages can be polarized in culture with specific cytokines, this suggested these states may be important to disease [12, 119–121]. However, both *Arg1* and *Nos2* are rarely expressed by microglia in MS models and do little to predict the microglia phenotype [80, 90, 120]. Monocyte-derived macrophages can express these markers differentially, but also take on several other cellular phenotypes suggesting greater diversity [122]. How the function of microglia and macrophages relate to their cellular phenotypes during MS remains an open question.

The identity of BAM-specific markers can be used to distinguish BAMs from microglia. Like microglia, BAMs are highly plastic and change their expression patterns during disease, so they may be confused with reactive microglia [123, 124]. Only recently are new markers becoming available, so the exact roles of BAMs are not as well characterized [71, 72, 125]. Studies demonstrated that BAMs are involved in recruiting immune cells to sites of inflammation and scavenging debris [126, 127]. BAMs are located at the CNS border and help regulate immune cell entry and neuroinflammatory responses within the leptomeningeal space [128]. BAMs during neuroinflammation upregulate expression of molecules such as MHC class II and CD40 that are involved in antigen presentation or co-stimulation, suggesting that they may regulate the activity of lymphocytes in the leptomeningeal space, perivascular space, or choroid plexus [84, 129, 130]. However, experiments that eliminated the MHC class II antigen presenting capabilities of microglia and BAMs found that these cells are not required for T cell-mediated EAE pathogenesis [124, 131–133]. Instead, it may be the antigen presenting capabilities of CNS-infiltrating myeloid cells, namely dendritic cells, that are involved in reactivating myelin-reactive encephalitogenic T cells towards a pro-inflammatory phenotype, thus inducing pathology in EAE. Overall, because most neuroimmunology studies have not made a distinction between BAMs, microglia, and infiltrating peripheral monocyte-derived macrophages, the exact contribution of BAMs to immune homeostasis of the normal CNS or MS pathophysiology is unclear.

### CNS macrophages in MS pathophysiology

Microglia and macrophages are the major cell types in MS lesions [58, 134]. While they predominantly have a pro-inflammatory phenotype in active lesions indicated by expression of inflammatory markers such as CD40, approximately 70% also express more homeostatic markers [135]. In both human MS and mouse models, microglia and macrophages in early CNS lesions produce increased amounts of inflammatory factors such as nitric oxide (NO), TNF-α, and IL-1β. As they continue to phagocytose, process, and clear cholesterol-rich myelin debris, they acquire a lipid-laden foamy phenotype that generally have more reparative anti-inflammatory characteristics [120, 121, 136]. Myelin breakdown in phagocytes is thought to generate lipid and cholesterol metabolites that bind and activate liver X receptors (LXR), which modulates inflammation and helps facilitate the reverse cholesterol transport system in microglia and macrophages [137–139]. Effective LXR-mediated cholesterol efflux also increases production of immunomodulatory factors such as IL-10 [136]. The ability of microglia and macrophages to efflux cholesterol-containing myelin debris decreases with age in mice [140]. The resulting cholesterol build-up forms crystals, leading to rupture of the lysosomes and activation of the inflammasome. Consequently, heightened microglia and macrophage inflammasome activity impairs remyelination in the CNS. Given that heighted inflammasome activity promotes neurodegeneration in other neurodegenerative conditions, the impaired efflux of cholesterol and subsequent inflammasome activation in microglia and macrophages may contribute to disability progression in PwMS [136, 140–142].

#### CNS macrophages in the MS brain

**Fig. 1:**
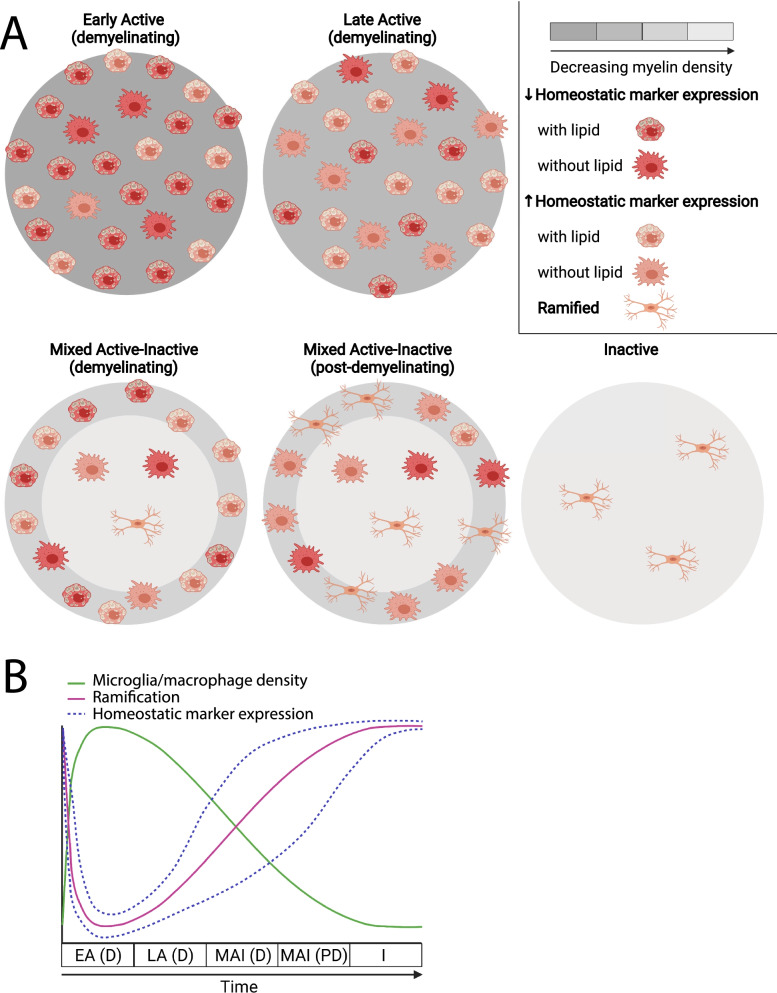
Summary of microglia and macrophages in different types of MS lesions. **A** Lesions are colored where a lighter grey indicates decreased myelin density. Reactive microglia and macrophages are depicted with large cell bodies and are categorized where red indicates decreased, and orange indicates increased expression of what are typically considered homeostatic markers such as P2RY12 or CD163. Reactive microglia and macrophages are also categorized based on whether they contain lipid-rich myelin degradation products. Non-reactivated microglia and macrophages are depicted with small cell bodies and a ramified morphology. **B** Patterns of microglia and macrophage characteristics (density, ramification, and homeostatic marker expression) over time in different MS lesion types. *EA (D)* early active (demyelinating), *LA (D)* late active (demyelinating), *MAI (D)* mixed active–inactive (demyelinating), *MAI (PD)* mixed active–inactive (post-demyelinating), *I* inactive. For **A** and **B**, active (post-demyelinating) lesions are not depicted due to limited histopathological assessments of microglia and macrophage characteristics

Study of CNS tissues from PwMS has enabled detailed pathological characterization of CNS macrophages in the MS brain. MS lesions have historically been characterized as active, chronic, or inactive based on the extent of demyelination. However, a more recent classification has proposed guidelines to distinguish lesions as active, mixed active–inactive, or inactive not only based on the presence or absence of ongoing demyelination, but also based on the characteristics of microglia and macrophages within the lesion [58]. The characteristics of microglia and macrophages in different MS lesion types are summarized in Fig. 1A, B. Most studies of MS lesions have used markers common to monocyte-derived macrophages, BAMs, and microglia such as IBA-1 and CD68. Markers such as TMEM119 provide better differentiation between microglia and recruited macrophages in MS lesions, although research groups have demonstrated *Tmem119* expression in microglia is reduced as they become reactive [84, 90, 143]. Recent advancements in single-cell RNA sequencing have provided a better framework for differentiating BAMs and microglia using their gene expression patterns [70, 124]. However, the exact proportions of microglia versus macrophages in MS lesions is largely undefined, because these cells are highly plastic and there are no pathological studies that use new markers to distinguish BAMs from microglia.

#### Lesion composition

##### Active lesions

Active lesions are mostly found in people with RRMS and shorter disease durations, and they can also be found in people with SPMS and PPMS with attacks and shorter disease durations [48, 58]. Active lesions are characterized by extensive demyelination and immune cell infiltration [58, 144]. Infiltrates contain T cells and B cells, but lipid-containing foamy microglia and macrophages are the major cell types in active lesions [37, 48, 58, 134]. These activated microglia and macrophages can be closely associated with or engulfing damaged transected axons [145]. Active lesions are present in the white matter, but they are also prominent in the grey matter [58]. In the cerebral cortex, active lesions are frequently associated with meningeal inflammation, and lesion rims often have high densities of activated microglia and macrophages containing myelin degradation products [37, 146, 147]. Haider et al. observed most neurons in these active cortical lesions had signs of oxidative damage [146]. In the deep grey matter, active lesions contain more inflammatory inducible nitric oxide synthase (iNOS)-positive microglia and macrophages, which are potential sources of oxidative damage. Microglia and macrophages form borders around deep grey matter lesions, although to a lesser extent than white matter lesions [148].

Active lesions can be subdivided based on the stage of demyelination [58]. Early active lesions are partially demyelinated, they have ongoing demyelination, and they contain microglia and macrophages with major (larger) and minor (smaller) myelin proteins [48, 58, 149]. The number of early active lesions declines quickly with disease duration [48]. Microglia and macrophages in these lesions have a less homeostatic phenotype. For example, early active lesions contain microglia with no expression of P2RY12, a marker that is only expressed by homeostatic microglia in rodents [79, 84]. Microglia and macrophages closely associated with damaged axons produce high levels of glutamate, indicated by high expression of the glutamate synthesizing enzyme, glutaminase, which is not expressed by microglia in normal control white matter [144].

Late active lesions are more demyelinated than early active lesions, they still have ongoing demyelination, and they contain microglia and macrophages with only major myelin proteins, which take longer to clear than minor myelin proteins [48, 58, 149]. Compared to early active lesions, microglia and macrophages in late active lesions transition to a more intermediate phenotype with both pro- and anti-inflammatory characteristics [84, 135, 150]. Kuhlmann et al. also classified a subset of active lesions that no longer have ongoing demyelination as active and post-demyelinating [58]. These lesions are heavily infiltrated by microglia and macrophages that lack both major and minor myelin proteins [58, 134].

##### Mixed active–inactive lesions

Mixed active–inactive lesions are most common in people with progressive MS with attacks or a disease duration longer than 10 years [48, 58]. These lesions, also commonly referred to as “chronic active”, have an inactive demyelinated center defined by fewer reactive microglia and macrophages and a higher density of microglia and macrophages at the lesion rim [48, 58, 84, 145]. Mixed active–inactive lesions can also be classified as demyelinating or post-demyelinating [58]. Mixed active–inactive demyelinating lesions, also called “smoldering” or “slowly expanding”, have ongoing myelin loss at the lesion rim with microglia and macrophages that contain myelin degradation products [48, 58]. The rims of mixed active–inactive post-demyelinating lesions have microglia and macrophages that lack myelin degradation products [58]. Microglia and macrophages in the center of mixed active–inactive lesions generally have a pro-inflammatory phenotype as they have high expression of glutaminase and low expression of immunomodulatory markers such as P2RY12 and CD163—a scavenger receptor that enhances phagocytosis and repair [84, 135, 144, 150]. Some research groups have found microglia and macrophages in the lesion border express lower levels of glutaminase and are more likely to express markers that indicate an immunomodulatory phenotype such as CD163 compared to those in the lesion center [144, 150]. However, other studies of mixed active–inactive lesions indicate borders tend to have increased expression of markers more characteristic of pro-inflammatory microglia and macrophages such as CD40, CD64, CD32, and iNOS and have reduced expression of more homeostatic markers such as CD163 [84, 134, 135].

##### Inactive lesions

Inactive lesions are most common in people with SPMS without attacks or a disease duration longer than 15 years [48, 58]. Inactive lesions are extensively demyelinated, have clear borders, and no ongoing myelin loss based on the absence of myelin debris within microglia or macrophages [58, 151]. The microglia and macrophage density is lower or similar compared to normal white, grey, and deep grey matter controls [48, 58, 84, 148, 152]. These microglia and macrophages have a predominantly surveillant ramified morphology, they contain much less degraded myelin products, and some express homeostatic microglia markers such as P2RY12. Glutaminase expression is absent from microglia and macrophages in inactive lesions [37, 84, 144, 151].

### CNS macrophages in normal appearing CNS

Even though lesions are the major pathological hallmark of MS, PwMS also have alterations in the NAWM and NAGM compared to controls. The NAWM and NAGM is not associated with a greater density of microglia and macrophages in people with progressive MS compared to normal age-matched controls [37, 84]. However, the brains of people with progressive MS have significantly increased microglia reactivity in the NAWM and NAGM based on reduced expression of homeostatic microglia markers including P2RY12 compared to normal controls; microglia reactivity increases with disease duration. Increased microglia reactivity in MS is associated with more diffuse NAWM injury including myelin loss and axonal damage [41, 43, 58, 84]. Clusters of reactive microglia termed microglia nodules may be present in over half of PwMS, and they are found in areas around plaques and in NAWM in progressive MS, but not normal controls [41, 151, 153]. These nodules have been associated with Wallerian degeneration of axons in early MS lesions, and may even occur prior to demyelination [153, 154].

### Identifying increased CNS macrophage reactivity in the progressive MS brain

Recent advancements in positron emission tomography (PET) imaging allow the visualization of radioligand binding to 18 kDa translocator protein (TSPO), which is a protein that is thought to be upregulated in reactive microglia and macrophages [155, 156]. PET studies have found increased binding of the first generation radioligand ^11^C-PK11195 to TSPO in the NAWM, thalamus, and cortical grey matter of PwMS, particularly SPMS, compared to healthy controls [156–159]. The increase in total ^11^C-PK11195 binding in the cortex correlated with increased expanded disability status scale (EDSS) disability scores and progression in SPMS but not RRMS [156, 157]. Second generation radioligands such as ^11^C-PBR28 with higher binding specificity and affinity for TSPO showed similar increases in signal, particularly in SPMS, that was associated with increased neurological disability and progression [160, 161]. People with SPMS also have greater overall TSPO expression throughout the grey matter compared to those with RRMS [161]. One limitation of TSPO-PET imaging is that TSPO may not be specific only to reactive microglia as it is also expressed in some reactive astrocytes and vascular endothelial cells [155, 162]. TSPO expression also does not differentiate between human microglia stimulated in vitro under pro-inflammatory or anti-inflammatory conditions [163]. As a result, there is a need for more microglia-specific TSPO-PET radioligands that can distinguish between inflammatory and homeostatic microglia phenotypes [39].

Based on TSPO-PET findings, microglia and macrophage reactivity occurs early in the disease course of MS and escalates during progressive MS. TSPO-PET based microglia and macrophage reactivity predicts disease progression suggesting that these cells have important roles in neurodegenerative processes, particularly during progressive disease.

## Microglia and macrophage role in neurodegeneration

The etiology and mechanisms of neurodegeneration in MS remains an area of intense investigation. Over the years two paradigms have emerged to explain the etiology of MS, the outside-in and the inside-out paradigms [164, 165]. The outside-in theory suggests that MS begins with CNS-targeted autoimmunity resulting in secondary neurodegeneration. On the other hand, the inside-out theory proposes that neurodegeneration is the primary event resulting in a secondary autoimmune response. Despite a century of studying MS, the true nature of the disease remains unknown. Much of what we know about neurodegeneration comes from cross sectional analysis of MS tissue and imperfect animal models of progressive MS creating a significant challenge in understanding the underlying mechanisms. For example, the EAE model is biased towards an autoreactive CD4 T-cell response while cytotoxic CD8 T cells are known to contribute to MS pathology. Given that many extracellular factors regulate microglia and macrophage enrichment and reactivity during all disease stages, many mechanisms may define their contribution to neurodegeneration in progressive MS including the release of neurotoxic factors that may result in accumulation of mitochondrial injury and sustained pro-inflammatory cytokine production.

### Extracellular factors regulating microglia and macrophages

**Fig. 2:**
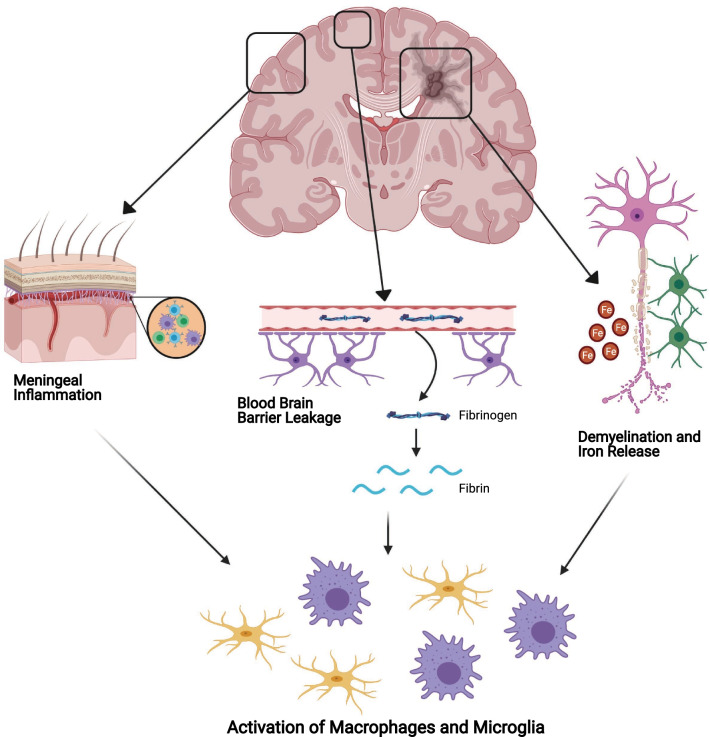
Extracellular factors regulating microglia and macrophage reactivity in progressive MS. Many characteristic features of MS pathophysiology may lead to the microglia and macrophages reactivity. Accumulation of meningeal inflammation, including accumulation of B cells, T cells and macrophages, results in microglia and macrophages reactivity in the underlying cortex. Leakage of the BBB results in blood components such as fibrinogen leaking into the CNS. Once in the CNS, fibrinogen is converted to fibrin which acts as a potent stimulus of microglia and macrophages. Demyelination and death of iron-rich oligodendrocytes release excess iron into the extracellular space, which can in turn act as a stimulus for microglia and macrophages

Characteristic features of progressive MS pathophysiology, including iron deposition, involvement of fibrinogen, and meningeal inflammation are likely important contributors to the reactivity of microglia and macrophages, potentially contributing to neurodegeneration (Fig. 2).

#### Iron

Iron is essential in the normal functioning of the CNS and is highly enriched within myelinating oligodendrocytes [166–168]. Iron concentrations increase within the brain parenchyma during normal aging, but this is accelerated in PwMS and is more pronounced in those with progressive disease courses [169, 170]. Iron levels within the brain are normally buffered by proteins such as ferritin, which holds the non-toxic ferric form (Fe^3+^) of iron and is found within oligodendrocytes and microglia [32]. The release of excess iron during CNS demyelination is potentially directly neurotoxic due to increased extracellular concentrations of unbuffered ferrous iron in the form of divalent cations (Fe^2+^), which amplifies oxidative injury via the Fenton reaction [171]. Unbuffered iron can also induce toxicity indirectly in culture as demonstrated by FeSO_4_ (a Fe^2+^ donor) stimulated microglia producing ROS at levels comparable to lipopolysaccharide (LPS—a TLR2/4 agonist from gram negative bacteria) stimulated microglia [169]. The presence of FeSO_4_ also exacerbated LPS-induced microglia-dependent neuronal loss [169]. The neuronal toxicity induced by iron and LPS-stimulated microglia is prevented by inhibiting the ROS producing enzyme, NADPH oxidase 2 [169], suggesting a potential role of iron in microglia mediated neurodegeneration. Iron is also immunomodulatory. Iron-deficient mice failed to develop EAE, while iron overloaded mice developed typical disease [172], suggesting that iron is an important regulator of immune-mediated neurotoxicity.

Analysis of MS tissue by MRI demonstrates that there is increased iron concentrations at the macroscopic level, specifically in the deep grey matter structures [167, 171, 173]; these are the same structures that display atrophy even early in PwMS [32]. Following demyelination there is a shift in the distribution of iron; iron is increased in microglia and macrophages in areas surrounding plaques [174], but reduced within lesions [148, 175, 176] and the NAWM of PwMS [177–179]. The source of increased iron in microglia and macrophages is multifactorial and includes the death and damage to iron-rich oligodendrocytes, vascular damage, and infiltration of peripheral immune cells involved in systemic iron homeostasis [180]. Iron analysis by MRI has increased our knowledge of the pathophysiology of MS, but tools such as magnetic resonance microscopy (MRM) are addressing the disconnect between small scale pathology of individual cells and how this correlates with MRI data [181]. Using MRM, Nair et al. visualized iron containing cells from post-mortem MS tissue and found that changes in iron accumulation within cells—mainly decreased iron within oligodendrocytes—give rise to the differential iron expression seen between cortical lesions and normal cortex [181]. Taken together, this increased iron storage in microglia and macrophages or heightened free ferrous iron may contribute to ongoing neurodegeneration in MS by amplifying oxidative stress.

#### Blood–brain barrier leakage

Leakage of the BBB occurs during inflammation to permit leukocyte entry into the CNS, but lower levels of BBB leakage happen as a natural consequence of aging [182]. Disruption of the BBB allows elements from the blood—not normally in the CNS—to enter such as fibrinogen. Fibrinogen entering into the CNS parenchyma is converted into insoluble fibrin, which is a potent stimulus for microglia and macrophage reactivity [183–185]. In response to fibrin, microglia surround leaky blood vessels and increase production of ROS [186]. A study by Yates et al. demonstrated increased extracellular fibrin/fibrinogen deposition in the cortex of people with progressive MS compared to healthy controls. Interestingly, heightened levels of extracellular fibrin/fibrinogen was associated with reduced neuronal density, but not demyelination [187]. Reactive microglia surrounding fibrin deposits are observed in early MS and EAE disease, associated with axonal damage, and found prior to the formation of demyelinated lesions and T-cell infiltration [188]. Depletion of fibrin in animal models of MS resulted in reduced inflammation, reactive microglia, demyelination, and axonal damage, suggesting the importance of protecting against fibrin-induced toxicity in PwMS [186, 189].

#### Meningeal inflammation

Widespread inflammation within the meningeal layers of the brain and spinal cord is found in all MS disease courses [190]. The formation of ectopic follicle-like structures in the meninges was found in 40% of SPMS cases [44]. The inflamed meninges contain macrophages and T cells, and the density of these cells was strongly associated with the microglia and macrophage density in the underlying subpial parenchyma [42, 191]. Indeed, the amount of meningeal inflammation correlates positively with cortical demyelination and neurodegeneration. The pattern of demyelination, neuronal injury, and axonal loss is greatest in the outer cortical layers and less pronounced in inner layers, suggesting that meningeal associated pro-inflammatory cytokine or neurotoxic factor radiate into the superficial grey matter to drive neurodegeneration [41–44, 46, 192]. These factors may induce direct toxicity or stimulate an indirect response in the CNS parenchyma that is degenerative.

Meningeal inflammation can induce neurotoxicity in the cortex and the underlying white and grey matter [44, 192, 193]. Meningeal transduction of lentiviruses producing IFN-γ and TNF-α cytokines induces demyelination, reactive microglia, neuronal loss, and upregulates genes related to necroptosis in the underlying grey matter [194]. Therefore, meningeal inflammation is sufficient to induce demyelination and neurodegeneration. Meningeal inflammation induces a phenotypic changes in cortical microglia, and one phenotype characterized by low levels of homeostatic microglia markers P2Y12 and TMEM119 was associated with substantial neuronal loss [195]. How meningeal inflammation drives neurodegeneration and whether it is via microglia and macrophage mediated mechanisms during progressive MS is still an open question.

### Release of neurotoxic factors by microglia and macrophages

#### Reactive oxygen and reactive nitrogen species

The direct release of neurotoxic factors including ROS or RNS, which are more pronounced in progressive MS, are one potential mechanism of microglia and macrophage mediated neurodegeneration in progressive MS [196]. In culture, when microglia are stimulated with LPS, they respond by increasing the production of ROS and RNS [197–199]. The main enzyme responsible for this ROS production for microglia is NADPH oxidase [197, 198]. Indeed, when microglia are cultured with immature oligodendrocytes and treated with LPS, there is a dramatic loss of immature oligodendrocyte that is prevented by inhibiting NADPH oxidase, suggesting that microglia ROS can be toxic in culture [197]. When treated with LPS, microglia also upregulate iNOS and produce RNS in culture [200, 201], but recent studies in vivo found that macrophage iNOS expression predominates, with limited microglial iNOS expression during EAE [90, 120, 202]. During EAE a greater proportion of macrophages than microglia also generate ROS, but both ROS-associated microglia and macrophages share a core oxidative stress signature [202]. The production of ROS by phagocytes during EAE results in injury to myelin and axons and is diminished with ROS and RNS scavengers [102, 203].

**Fig. 3:**
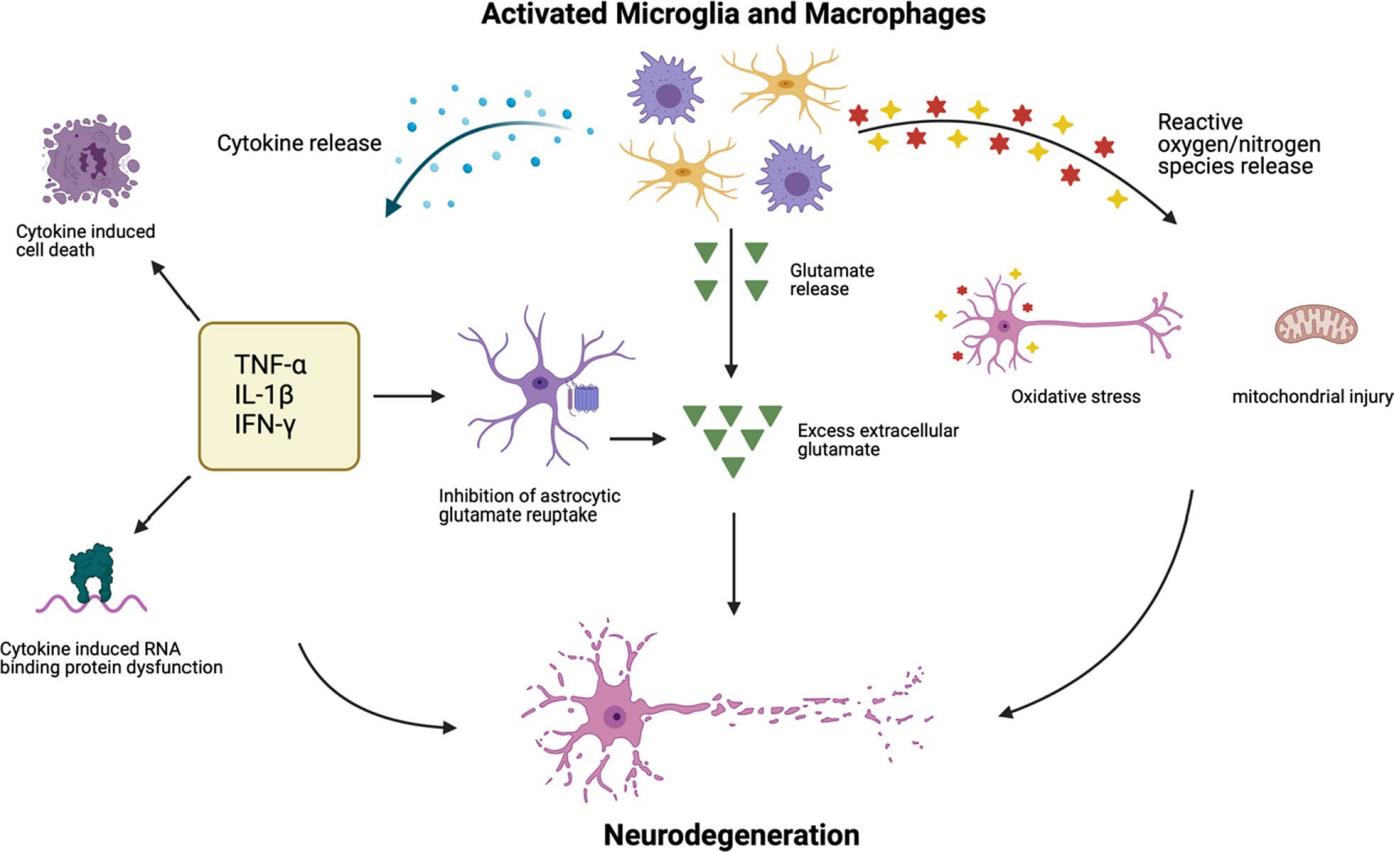
Microglia and macrophage mediated mechanisms of neurodegeneration in progressive MS. Microglia and macrophages release many cytokines, including TNF-a, and IL-1β, which may contribute to neurodegeneration via cytokine induced cell death, inhibition of astrocytic glutamate reuptake, and via the induction of dysfunctional RNA binding proteins. Microglia and macrophages can also release glutamate, potentially contributing to glutamate excitotoxicity and neurodegeneration. Lastly, microglia and macrophages release ROS/RNS which may contribute to neurodegeneration by inducing oxidative stress and mitochondrial injury

ROS and RNS can also contribute to neurodegeneration by inducing mitochondrial dysfunction in neurons that persists and accumulates over time [204]. In progressive MS, deep cortical neurons contain mutations within mitochondrial DNA [205]. MS lesions have significant mitochondria disturbances including decreased expression of electron transport chain complex I, III and IV [206–208], which correlates with axonal damage [209]. The corresponding energy deficiencies due to mitochondrial dysfunction amplify oxidative stress via the release of more oxygen radicals into the CNS [204]. Considering the high energy consumption the brain utilizes, impaired energy production due to mitochondrial dysfunction likely contributes to neurodegeneration. As a strategy to protect mitochondria from ROS—especially given the oxidative damage within MS lesions and within NAWM [210]—strategies that mitigate oxidative stress or reduce ROS production by microglia and macrophages are likely an important neuroprotective strategy for MS (Fig. 3).

#### Glutamate release

Glutamate is an excitatory neurotransmitter playing an important role in neuronal signaling, however when produced in large quantities it becomes toxic, resulting in damage to both neurons and oligodendrocytes [211–215]. Increased CNS glutamate was found prior to demyelination in PwMS [216], suggesting that it may be a precursor to the formation of MS lesions. A study looking at cortex specimens from predominantly progressive MS cases found loss of glutamate reuptake mechanisms in astrocytes in the presence of reactive microglia [217]. Reactive microglia release cytokines such as TNF-α, which reduces astrocytic glutamate uptake through a pathway involving the release of NO, thus increasing extracellular glutamate concentrations [218]. This increased extracellular glutamate is associated with demyelination and neuronal damage [217]. Increased extracellular glutamate in the CNS can also be a result of reactive microglia and macrophages, energy deficiencies, increased oxidative stress, and mitochondrial dysfunction [219]. Microglia can release glutamate via the cystine-glutamate antiporter system X_c_^−^ [220, 221]. For this antiporter system, cystine is imported and serves as a co-factor for the antioxidant glutathione—potentially to serve as an antioxidant for the microglial oxidative bursting [222]—resulting in the release of glutamate [223]. In culture, microglia release glutamate if supplemented with cystine or stimulated with LPS or the pro-inflammatory cytokines IFN-γ and TNF-α [220, 221, 224]. Microglial glutamate release is toxic to oligodendrocytes, which can be prevented by either treatment with glutamate receptor or system X_c_^−^ transporter antagonists [220, 221].

Despite the release of toxic levels of glutamate by microglia in culture, less is understood about glutamate release from microglia and macrophages in vivo. Injections of LPS with cystine in vivo is more toxic to neurons than LPS alone suggestive of system X_c_^−^ transporter toxicity [225] and system X_c_^−^ transporter antagonists are protective against EAE [220]. The removal of one glutamate receptor subunit (the GluA4 from AMPA receptor) from oligodendrocytes protects them during EAE, suggesting an ongoing glutamate toxicity during EAE [226]. However, T cells can release glutamate during EAE [227] and the system X_c_^−^ transporters are expressed by astrocytes [228] and leukocytes [229]. The contribution of microglial glutamate is, therefore, still unclear as is the contribution of glutamate toxicity in later stages of progressive MS.

### Sustained pro-inflammatory cytokine production

In progressive MS there is increased microglia and macrophages reactivity into a pro-inflammatory phenotype, which may contribute to neurodegeneration via a number of potential mechanisms. Pro-inflammatory microglia activation results in a loss of immunosuppressive factors including CX3CR1 and CD200 and increased secretion of the pro-inflammatory cytokines IL-1β, IL-6, and TNF-α among other neurotoxic factors [230]. Given that microglia and macrophages express and release many cytokines in MS, individual or combinations of cytokines may induce neuronal toxicity.

#### TNF-α

TNF-α is elevated in CSF of people with progressive MS compared to both RRMS and healthy controls [111]. TNF-α is expressed by microglia and macrophages during EAE and MS [90, 193, 202]. In culture, TNF-α induces oligodendrocyte cell death by necroptosis, a form of programmed necrosis that releases inflammatory molecules [231, 232]. Necroptosis is controlled by a protein cascade consisting of receptor-interacting seine/threonine-protein kinase 1 and 3 and phosphorylated mixed lineage kinase domain-like pseudokinase; TNF-α stimulated oligodendrocyte cell death was attenuated in the presence of a RIPK1 inhibitor [231]. In both the EAE and cuprizone models of MS, a RIPK1 inhibitor attenuated oligodendrocyte death and improved disease outcomes [231]. TNF-α can activate tumor necrosis factor receptor 1 (TNFR1) resulting in a pro-inflammatory response inducing necroptosis, or, alternatively, stimulate tumor necrosis factor receptor 2 (TNFR2) promoting a protective response [233]. People with progressive MS have elevated TNF-α expression in the cortex and meninges which shifts the TNF receptor expression balance from a TNFR2 cell survival to TNFR1 cell death [193, 231]. TNFR1 signaling is enhanced within cortical and subpial lesions associated with meningeal inflammation, and this signaling is linked to an upregulation of key necroptotic pathway regulators within neurons, oligodendrocytes, and microglia [231, 233, 234]. Therefore, TNF-α may be inducing necroptosis of neurons and oligodendrocytes within the CNS. Inhibition of soluble TNF-α, which has been found to signal via TNFR1 reduced the clinical severity of EAE while preserving axonal integrity and promoting remyelination [235]. Selective inhibition of TNFR1 ameliorated EAE symptoms in both prophylactic and therapeutic treatments. Unselective anti-TNF therapy in PwMS resulted in a significantly increased risk of disease exacerbation, future therapies directed towards selective TNFR1 inhibition may prove to be beneficial in progressive MS [236].

#### *IL-1*β

People with progressive MS have increased CSF and serum concentrations of IL-1β compared to both RRMS and healthy controls [110, 237]. Similar to TNF-α, addition of IL-1β in culture caused oligodendrocyte cell death [231, 232] and IL-1β is enriched in microglia, macrophages, and neutrophils during EAE [90, 202, 238]. The loss of IL-1β reduces EAE susceptibility threefold, which is consistent with the pathogenic nature of this cytokine [238]. However, during EAE, IL-1β also interacts with barrier cells such as endothelial cells to alter leukocyte trafficking and damage may not be a result of direct IL-1β toxicity [238, 239]. Given that cytokines are instrumental in regulating trafficking into the CNS, it is challenging to determine whether the release of cytokines by microglia and macrophages induce direct toxicity in the CNS, or indirectly provoke toxicity by recruiting pathogenic leukocytes. For example, the overexpression of granulocyte–macrophage colony-stimulating factor in peripheral helper T cells, which is minimally expressed by microglia or macrophages during EAE [240], recruits ROS-associated macrophages into the CNS that induce toxicity [241]. Combinations of cytokines can also induce toxicity indirectly via astrocytes, for instance, IL-1β, TNF-α and C1q induce a neurotoxic astrocyte phenotype [242]. Taken together, disturbances in cytokine signaling can result in microglia reactivity and damage to neurons and oligodendrocytes, serving as a potential point of therapeutic intervention.

#### Cytokine induced RNA binding protein dysfunction

The CSF of people with progressive MS contains high levels of a number of pro-inflammatory cytokines including TNF-α and IFN-γ [110, 243, 244], which can result in the mislocalization of RNA binding proteins (RBP) within neurons and oligodendrocytes. Addition of TNF-α and IFN-γ in culture induce RBP mislocalization and dysfunction [245, 246]. RBPs are responsible for maintaining RNA metabolism, including RNA transport, splicing, and stability. A single RBP is capable of modulating the expression and function of multiple target RNAs, thus the dysfunction of a single RBP disrupts the regulation of many downstream RNAs. RBP dysfunction is characterized by mislocalization of these proteins from their homeostatic nuclear location to the cytoplasm; in severe cases RBPs are completely absent from the nucleus [246–249]. RBP cytoplasmic accumulation results in the formation of toxic aggregates of RNAs and proteins in addition to the loss of normal RBP functioning in the nucleus, which causes impaired RNA metabolism.

RBP dysfunction contributes to neurodegeneration in a number of diseases including MS [248–253]. The RBPs heterogenous nuclear ribonucleoprotein A1 (hnRNP A1) and transactive response DNA binding protein 43 (TDP-43) are mislocalized within the ventral spinal cord neurons during chronic EAE, which is correlated with axonal damage and neuronal loss [246, 247, 254]. Indeed, RBP cytoplasmic mislocalization and nuclear depletion are also present in cortical neurons and oligodendrocytes located in pathological specimens from PwMS [246, 254, 255]. Analysis of cortical neurons from 6 control cases and 12 MS cases (6 progressive MS, 1 RRMS and 5 unknown disease courses) demonstrated significant mislocalization of the RBPs hnRNP A1 and TDP-43, demonstrating that dysfunctional RBPs may contribute to neurodegeneration in progressive MS as in other neurological diseases [255]. Taken together, pro-inflammatory cytokines can cause RBP dysfunction in neurons and oligodendrocytes, potentially contributing to ongoing neurodegeneration. Given microglia and macrophages are an important source of pro-inflammatory cytokines and oxidative stressors, they may trigger RBP dysfunction in MS.

## Progressive MS therapies

### Disease-modifying drugs approved for progressive MS

**Fig. 4:**
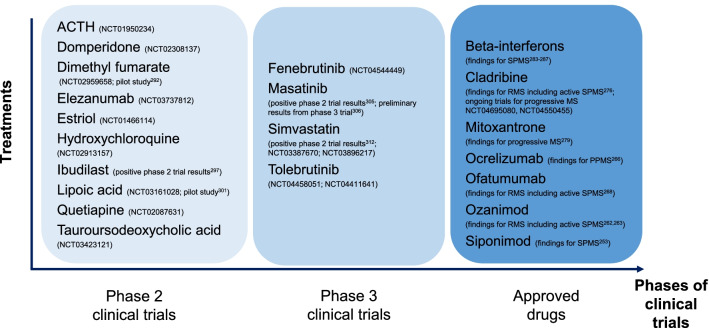
Phase 2/3 clinical trial of drugs and DMDs approved for progressive MS. The registration numbers with clinicaltrials.gov are shown in brackets for drugs currently being assessed in phase 2/3 clinical trials as potential treatments for progressive MS. The references for the published findings are also indicated in brackets (if applicable)

While there are over 15 DMDs approved for the treatment of RRMS over the last two decades, there are only limited therapeutic options currently available to treat progressive MS [4]. A range of beneficial and detrimental effects of microglia and macrophages have been reported in the models of MS [11, 15, 16], but drugs that solely target these innate immune cells are lacking. The recent treatment successes for progressive MS include sphingosine-1-phosphate (S1P) receptor modulator (siponimod), B-cell targeted therapy (ocrelizumab), and selective immune reconstitution therapy (cladribine) [256], while mitoxantrone and beta-interferon were the first few drugs used for treatment of SPMS. In this section, we described several approved DMDs for the treatment of progressive MS (Fig. 4).

#### S1P receptor modulators

Siponimod is a S1P receptor modulator approved by the United States Food and Drug Administration (FDA) in 2019 and was the first oral DMD indicated to treat SPMS with active disease [257]. In the phase 3 EXPAND study, siponimod reduced the risk of 3-month confirmed disability progression by 21% compared with placebo and slowed the rate of brain volume loss over 12 and 24 months in people with SPMS [258]. The main effects of siponimod are attributed to the functional antagonism of S1P1, which prevents the egress of peripheral lymphocytes from lymph nodes and diminishes their entry into the CNS [258, 259]. Siponimod binds selectively to the S1P1 and S1P5 receptors and these two subtypes of S1P receptors are also expressed in CNS-resident cells including microglia, astroglia, and oligodendrocytes [259]. Siponimod attenuated microglial release of the cytokines IL-6 and RANTES in cell culture and during EAE [259, 260]. IL-6 and RANTES are found in brain lesions and CSF of PwMS [261, 262]. Siponimod is currently only approved for active SPMS as the subgroup analyses from EXPAND study failed to show a statistically significant improvement in 3-month confirmed disability progression among people with no relapses in the previous 2 years [263].

Other S1P receptor modulators approved for the treatment of MS to date include fingolimod and ozanimod. Fingolimod (not selective for specific S1P receptors) [264] is currently indicated only for RRMS as its phase 3 clinical trial in progressive MS failed to demonstrate beneficial effects [265]. Ozanimod has similar target receptors (selective for S1P1 and S1P5) as siponimod and was recently approved by the FDA to treat relapsing forms of MS including active secondary progressive disease [266] based on results from phase 3 clinical trials (RADIANCE and SUNBEAM) [267, 268]. Ozanimod reduces microglia and macrophage pro-inflammatory cytokine expression, which may account its potential neuroprotective effects [269].

#### B-cell depletion

Ocrelizumab is a B-cell depleting agent, and is the first DMD indicated to treat both RRMS and PPMS [270]. The safety and efficacy of ocrelizumab in PPMS had been shown in a phase 3 clinical trial (ORATORIO). The risk of 12-week confirmed disability progression was reduced by 24% in the ocrelizumab-treated arm compared to placebo [271]. Another B-cell depleting agent, ofatumumab, was also recently approved to treat both RRMS and active SPMS in the United States [272] based on the results from phase 3 clinical trials (ASCLEPIOS I and II) showing lower annualized relapse rates in the ofatumumab-treated arm compared to the teriflunomide-treated arm [273]. B-cells are associated with meningeal inflammation that overlie demyelinating cortical lesions, which involve a rise in microglia reactivity [274]. Several open-label trials are underway to assess the treatment effect of ocrelizumab on CNS microglia reactivity as measured by TSPO-PET [275–277].

#### Immunosuppressants

Cladribine is a selective immunosuppressant and is approved to treat both RRMS and active SPMS in the United States [278]; it is only indicated for the treatment of highly active RRMS in Europe [279]. The safety and efficacy of cladribine had been examined in a phase 3 clinical trial (CLARITY) in people with RRMS [280], and a phase 2 clinical trial (ONWARD) in people with RRMS or SPMS with relapses [281]. The benefits of cladribine for the treatment of progressive MS remains to be explored in further clinical trials (ClinicalTrials.gov NCT04695080, NCT04550455). In addition to lymphocyte depletion, cladribine can cross the BBB and exert effects directly on CNS cells. For example, primary microglia cultures treated with cladribine reduced granularity, phagocytotic ability and altered gene expression of microglia suggesting a less activated phenotype [282, 283]. Cladribine also induces apoptosis in microglia cultures [282].

Mitoxantrone is an immunosuppressant and was the first drug approved for SPMS and progressive-relapsing MS based on the results shown in phase 3 trial (MIMS) [284, 285]. The primary outcome comprised five clinical measures including changes in the EDSS, ambulation index, standardized neurological status, number of treated relapses, and time to first treated relapse [284]. PwMS treated with high-dose mitoxantrone (12 mg/m^2^) improved on these five clinical measures compared to placebo over the short-term (2 years) clinical trial. However, there were concerns about the risk of cardiac dysfunction and acute leukemias with mitoxantrone treatment, as shown in studies with longer periods of follow-up [286]. The risk of cardiotoxicity also limits the long-term administration of mitoxantrone. Mitoxantrone can cross the BBB and is toxic to LPC-activated microglia in culture at high concentrations and at lower concentrations promotes the release of the immunoregulatory cytokine IL-10 [287].

#### Beta-interferon

The treatment of SPMS and PPMS with beta-interferon has mixed findings. Beta-interferon was used as the first-line treatment for SPMS given the worse risk–benefit profile with mitoxantrone [288]. The beneficial effects of beta-interferon on relapse-related outcomes (i.e., relapse rates) were shown in people with SPMS [289–292], while one out of the five studies found beneficial effects on short-term disability progression outcomes [288, 289]. La Mantia et al., following their systematic review of the literature, concluded that the anti-inflammatory effect of beta-interferon are not able to retard progression once it was established [288]. Similarly, clinical trials of beta-interferon in PPMS failed to demonstrate beneficial effect on disability progression outcomes [293, 294].

### Ongoing trials for progressive MS

There are several therapies being evaluated as a potential treatment option for progressive MS, such as immunomodulatory therapies that can target myeloid cells (e.g., dimethyl fumarate, ibudilast, lipoic acid) or as a potential neuroprotective agent (e.g., simvastatin). Other examples of drugs currently being assessed in phase 2/3 trials as potential treatments for progressive MS are listed in Fig. 4.

#### Dimethyl fumarate

Dimethyl fumarate is a nuclear factor (erythroid-derived 2)–like 2 (Nrf2) activator and is currently approved for the treatment of RRMS [295, 296]. A single-center observational pilot study showed that the EDSS score of over 75% of the 26 people with progressive MS treated with dimethyl fumarate remained stable or improved [297]. The safety and efficacy of dimethyl fumarate for use in PPMS is currently being assessed in a phase 2 trial (ClinicalTrials.gov NCT02959658), although another phase 2 trial of dimethyl fumarate in people with SPMS was terminated early by the pharmaceutical company (ClinicalTrials.gov NCT02430532). Activation of the Nrf2 antioxidant pathway by fumarates are neuroprotective in a chronic EAE model [298]. Nrf2, a target of dimethyl fumarate, is an important regulator of redox homeostasis and responding to ROS [299]; ROS are important contributors to axonal damage in both MS and animal models [300]. The synthesis of pro-inflammatory mediators such as iNOS, TNF-α, IL-1β and IL-6 are reduced in reactive microglia and astrocyte cultures pre-treated with dimethyl fumarate, suggesting that the neuroprotective effects of dimethyl fumarate may be attributed to its ability to inhibit expression of several neuroinflammatory mediators [301].

#### Ibudilast

Ibudilast is a non-selective phosphodiesterase inhibitor. Ibudilast reduced the progression of brain atrophy (i.e., about 2.5 mL less brain-tissue loss) in a phase 2 trial (NN102/SPRINT-MS) for the treatment of PPMS or SPMS compared with placebo [302]. However, its use was associated with gastrointestinal and other adverse effects. A larger phase III trial is needed to further examine the safety and efficacy of ibudilast for progressive MS. Ibudilast can cross the BBB, and inhibits macrophage migration inhibitory factor, a pro-inflammatory protein mainly secreted from microglia and macrophages [90, 302–304]. Ibudilast suppresses production of NO, ROS, IL-1β, IL-6, and TNF-α, as well as enhances production of immunomodulatory cytokines and neurotrophic factors in neuron and microglia cultures [305].

#### Lipoic acid

Lipoic acid is an antioxidant. The effects of lipoic acid on the annual percent change brain volume was assessed in a randomized controlled pilot trial [306]. Whilst there was a 68% reduction in annual percent change brain volume reported among people with SPMS treated with lipoic acid compared with those treated with placebo, the study was limited by small sample size (*n* = 27 in the lipoic acid arm and *n* = 26 in placebo) [306]. A phase II trial with over 100 participants is currently underway to examine the effects of lipoic acid on mobility and brain volume in progressive MS, and the results are expected to be available in 2021 (ClinicalTrials.gov NCT03161028). Given that microglia and macrophages predominate in producing oxidative stress during EAE [202] and MS [101], antioxidants may be one means to limit potential ROS-mediated toxicity. Lipoic acid inhibited microglia and macrophages reactivity and reduced the migration of T cells and monocytes across the BBB in EAE [307–309]. Lipoic acid also works by stabilizing the integrity of the BBB [309].

#### Masitinib

Masitinib is a selective tyrosine kinase inhibitor that provided therapeutic benefit for people with PPMS and relapse-free SPMS in a phase 2a clinical trial [310]. Preliminary results of the phase 3 clinical trial of masitinib were presented at the recent MSVirtual Conference 2020 and showed that the primary endpoint (changes in disability measured using the EDSS) was met, with reduced EDSS observed in the masitinib-treated arm compared to the placebo-treated arm [311]. The treatment effect was maintained for both the PPMS and non-active SPMS subgroups of the study population. Masitinib is the first tyrosine kinase inhibitor in this class of agents that targets the innate immune system by inhibiting mast cell activity [310, 311], that may contribute to EAE and MS [312]. Masitinib also inhibits CSF1R [313], a key receptor to promote microglia proliferation [314], and may therefore modulate innate immunity for people with MS. Positive results have also been reported from clinical trials of masitinib for the treatment of other neurological and inflammatory diseases such as rheumatoid arthritis and Alzheimer’s disease [315, 316].

#### Simvastatin

Simvastatin is an HMG-CoA reductase inhibitor, or statin, used for the management of hypercholesterolemia. Simvastatin when given in high dose (80 mg) showed a 43% reduction in annualized brain atrophy among people with SPMS compared with placebo in a phase 2 clinical trial (MS-STAT) [317]. Here, simvastatin treatment had a positive effect on frontal lobe function and physical health-related quality of life [318]. The effects of high-dose simvastatin on disability progression is currently being investigated in a phase 3 clinical trials with over 1000 people with SPMS (ClinicalTrials.gov NCT03387670; estimated study end date August 2023). The effects of statins on microglia function have been examined both in vitro (i.e., microglia cell lines) and in vivo (i.e., rat models) [319]. There are growing evidence suggesting that statins diminish pro-inflammatory mediators that regulate the microglia reactivity [319]. Statins may provide protection in the CNS of progressive MS by inhibiting microglia activation, restraining pro-inflammatory mediators such as TNF-α, IL-1β, IL-6, ROS, IFN- γ, COX-2, PGE2, and RNS, but also by promoting release of immunoregulatory cytokines such as IL-10 [319]. According to computational causal modelling, the beneficial effects of simvastatin in progressive MS might be independent of the change in serum cholesterol levels, suggesting that the upstream intermediate breakdown products of the cholesterol synthesis pathway may be involved [320].

### Treatment trials with negative results

Several other drugs have also been investigated for the treatment of progressive MS via neuroprotective and experimental approaches (e.g., amiloride, fluoxetine, riluzole) or remyelination promoting approaches (e.g., biotin, opicinumab), but showed negative results (Table 1). For example, although the effects of high-dose biotin (MD1003) for the treatment of progressive MS shown in previous studies (pilot study and a phase 2 clinical trial) were encouraging [321, 322], the most recent phase 3 clinical trial failed to demonstrate any significant differences in disease progression between biotin-treated and placebo-treated arms [323]. High-dose biotin is a co-factor for essential carboxylases, which may help support myelin repair by enhancing the production of energy in neuron and it is generally well tolerated [323, 324]. Some studies suggested that the exposure to biotin was associated with new disease activity [325, 326]. Similarly, in an adequately powered multi-centre, multi-arm, phase 2b clinical trial (MS-SMART) based in the United Kingdom published recently, no significant differences in annual percent change brain volume were found among people with SPMS treated with placebo compared with each of the three active treatments (amiloride, fluoxetine and riluzole) [327], despite the positive effects shown in earlier work (i.e., in animals and pilot studies) [328–333]. Other recent examples of drugs that had been studied but showed negative results are listed in Table 1.

**Table 1:** Studies with negative results based on most recent trial

Medication	Relevant studies
Amiloride	Positive results from open-label study (pilot study) [328]
	Negative results from phase 2b clinical trial [317]
Biotin (MD1003, high dose)	Positive results from three-centers study (pilot study) [321]
	Positive results from phase 2 clinical trial [322]
	Negative results from phase 3 clinical trial [323]
Dronabinol	Negative results from randomized controlled trial [336]
Fingolimod	Negative results from phase 3 clinical trial [265]
Fluoxetine	Negative results from single-center study (pilot study with inadequate power) [337]
	Negative results from phase 2b clinical trial [317]
Glatiramer acetate	Negative results from randomized controlled trial [338]
Idebenone	Negative results from phase 1/2 clinical trial [339]
Lamotrigine	Negative results from phase 2 clinical trial [340]
Laquinimod	Negative results from phase 2 clinical trial [341]
Lenercept	Negative results from phase 2 clinical trial [342]
Lithium	Negative results from open-label pilot study [343]
MBP8298 (dirucotide)	Positive results in subgroup of participants from phase 2 clinical trial [344]
	Negative results from phase 3 clinical trial [345]
MIS416	Negative results from phase 2 clinical trial from pharmaceutical company report [346]
Natalizumab	Positive results from phase 2 clinical trial [347]
	Negative results on sustained disability progression (positive results on upper-limb component of disability) from phase 3 clinical trial [348]
Opicinumab	Negative results from phase 2 clinical trial [349]
	Development of opicinumab has been halted by the pharmaceutical company (October 2020) [350]
Riluzole	Positive results from pilot study [332]
	Negative results from phase 2b clinical trial [317]
Rituximab	Negative results from phase 2/3 clinical trial [351]

## Conclusion

Microglia and macrophages are both beacons for ongoing damage in MS brains, but they are also key regulators or contributors for ongoing neurotoxicity. New tools are defining an unappreciated diversity of these cells [80, 334, 335], but it is still unclear how this diversity coincides lesion progression, grey matter damage arising from meningeal inflammation, or ongoing tissue atrophy. Given the presence of reactive microglia in conjunction with these key pathological features of progressive MS, it is assumed the role of microglia and macrophages are significant. Yet it is still not possible to know whether these cells are merely responding to pathological stimulus or driving neurotoxicity. Perhaps the loss of critical microglial functions contributes to progressive MS. These fundamental questions need answers to move the field of progressive MS forward. With much emphasis on the MS model EAE, which poorly models progressive MS, an expansion of MS models may help address these areas of research. In the clinical setting, numerous exciting clinical trials are ongoing, emphasizing the importance of treating progressive MS. With critical insight on the role of microglia and macrophages as they relate directly to MS pathogenesis new therapeutic targets are likely to be identified, fueling the pipeline for the benefits of people with progressive MS.

## Data Availability

Not applicable.

## Abbreviations

BAMs: Border associated macrophages
BBB: Blood–brain barrier
CAMs: CNS-associated macrophages
CIS: Clinically isolated syndrome
CNS: Central nervous system
CSF: Cerebrospinal fluid
DMDs: Disease-modifying drugs
EAE: Experimental autoimmune encephalomyelitis
EDSS: Expanded disability status scale
FDA: Food and Drug Administration
Gd: Gadolinium
HnRNP A1: Heterogenous nuclear ribonucleoprotein A1
IL: Interleukin
iNOS: Inducible nitric oxide synthase
LPS: Lipopolysaccharide
MRI: Magnetic resonance imaging
MRM: Magnetic resonance microscopy
MS: Multiple sclerosis
NAGM: Normal appearing grey matter
NAWM: Normal appearing white matter
NO: Nitric oxide
Nrf2: Nuclear factor (erythroid-derived 2)–like 2
OPCs: Oligodendrocyte progenitor cells
PET: Positron emission tomography
PPMS: Primary-progressive multiple sclerosis
PwMS: People with multiple sclerosis
RBPs: RNA-binding proteins
ROS: Reactive oxygen species
RRMS: Relapsing–remitting multiple sclerosis
SPMS: Secondary progressive multiple sclerosis
S1P: Sphingosine-1-phosphate
TDP-43: Transactive response DNA binding protein 43
TLR: Toll-like receptor
TNR: Tumor necrosis factor
TNFR1: Tumor necrosis factor receptor 1
TNFR2: Tumor necrosis factor receptor 2
TSPO: Translocator protein
